# Loperamide-Induced Torsades de Pointes

**DOI:** 10.7759/cureus.64427

**Published:** 2024-07-12

**Authors:** Binita Bhandari, Saima Siddique, Sahar Tahir, Michael G Link

**Affiliations:** 1 Hospital Medicine, University of Pittsburgh Medical Center (UPMC) Harrisburg, Harrisburg, USA; 2 Cardiology, University of Pittsburgh Medical Center (UPMC) Harrisburg, Harrisburg, USA

**Keywords:** loperamide, torsades de pointes, qtc prolongation, ventricular arrythmia, loperamide overdose, loperamide cardiotoxicity

## Abstract

Loperamide is a readily available, over-the-counter medication used to treat diarrhea. At therapeutic doses, loperamide exerts its effects mainly on the intestinal opioid receptors with minimal psychoactive effects; however, at supratherapeutic doses, it reaches central opioid receptors. With tighter regulations on opioid prescriptions, loperamide has emerged as a popular drug of abuse among opioid users. At supratherapeutic doses, loperamide can cause severe cardiac toxicity, resulting in wide QRS rhythms, severe bradycardia, prolonged QTc, polymorphic ventricular tachycardia, and cardiac arrest. We present the case of a 27-year-old female with a history of heroin abuse who suffered torsades de pointes resulting in cardiac arrest in the setting of a loperamide overdose.

## Introduction

Loperamide is a widely available and commonly used over-the-counter and prescription antidiarrheal agent that acts as a μ-receptor agonist in the circular and longitudinal muscles of the gastrointestinal tract, thereby reducing acetylcholine and prostaglandin release and subsequently leading to inhibition of peristalsis [1].

Since roughly 2008 and rapidly increasing through 2011, descriptions of loperamide misuse began to appear in various online recreational drug use forums. Users described opioid euphoric effects and attenuation of opioid withdrawal symptoms when loperamide was ingested in high doses (70-100 mg daily) [2,3]. Between 2010 and 2015, there was a 91% increase in the intentional nonmedical use of loperamide [4]. In the United States FDA’s Adverse Reporting System, 12,851 loperamide cases were reported from 1977 to 2018, out of which 1,204 were strongly associated with loperamide abuse, and 56% of the adverse events were serious, frequently cardiovascular in nature, and led to 252 fatalities [5]. Recently, reports have described dramatic QT prolongation, wide complex tachycardias, polymorphic ventricular tachycardias, and sudden cardiac death associated with loperamide abuse. However, loperamide-induced cardiac toxicity remains underrecognized among healthcare professionals [6].

## Case presentation

**Figure 1:**
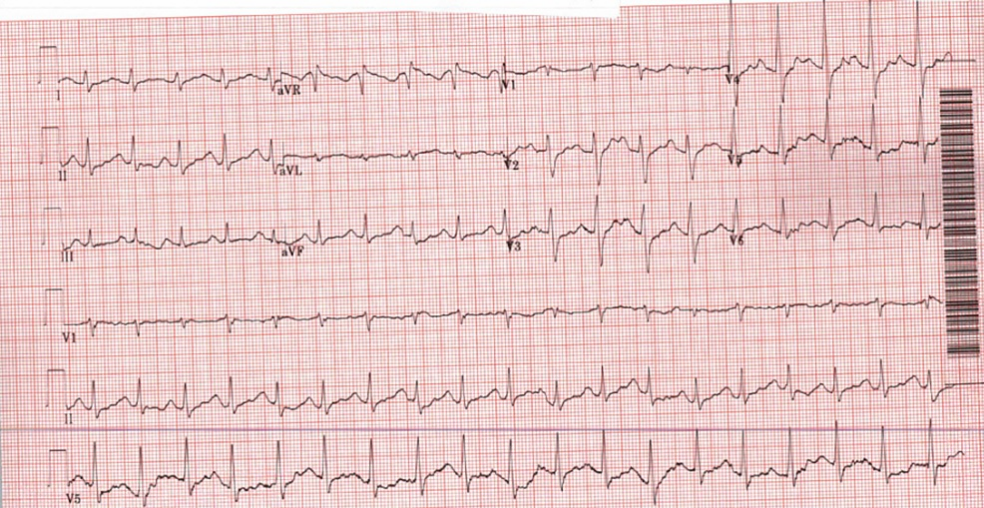
EKG on admission: sinus tachycardia with a QTc of 594 ms

A 27-year-old female with a past medical history of anemia and substance abuse (including heroin and loperamide) was found by her partner in an altered mental state with agonal breathing. A bottle containing loperamide pills was found by her side. The EMS gave her four intranasal spray doses of 0.2 mg of naloxone, following which minimal improvement in her breathing was noted. An endotracheal intubation was attempted but was unsuccessful, and she was brought to the ED by the EMS with bag and valve mask ventilation. In the ED, she was noted to be tachypneic and in distress with a respiratory rate of 37/min, along with a temperature of 36.7 °C, a heart rate of 76 beats/minute, and a blood pressure of 110/76 mmHg. She was intubated in the ED due to concerns about her inability to protect her airway. Laboratory investigations were notable for hemoglobin levels of 8.7 mg/dl, and a urine drug screen was positive for benzodiazepines. Her thyroid-stimulating hormone was within normal limits. An EKG revealed a corrected QT interval (QTc) of 594 ms (Figure 1). Chest X-rays showed findings consistent with multifocal pneumonia. She was admitted to the hospital and started on broad-spectrum antibiotics (vancomycin, cefepime, and metronidazole). She was also started on a bicarbonate drip, as recommended by toxicology, to narrow the QTc interval and prevent arrhythmias.

**Figure 2:**
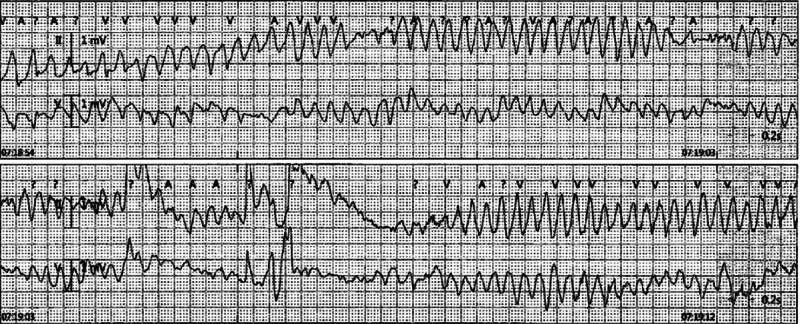
Telemetry strip that demonstrated torsades de pointes

A repeat EKG done after 24 hours showed an improved QTc of 405 ms. Her antibiotics were changed to azithromycin, which was discontinued after 48 hours as the patient developed two episodes of torsades de pointes. Despite receiving aggressive magnesium repletion, the patient continued to have frequent arrhythmias on telemetry and was started on isoproterenol. On day 4 of hospitalization, the patient developed a persistent polymorphic VT episode/torsades de pointes (Figure 2), which required four minutes of CPR, including one defibrillation shock. An EKG done following the return of spontaneous circulation showed an increased QTc interval of 565 ms. She was administered a lidocaine bolus and started on a lidocaine drip. An echocardiogram showed a normal ejection fraction of 55-60% with no regional wall motion abnormalities.

The patient was evaluated by an electrophysiologist and started on mexiletine. The isoproterenol and lidocaine drips were slowly tapered down and eventually stopped after two days. Her clinical course thereafter continued to improve, and she was subsequently discharged with a plan to follow up as an outpatient for further evaluation of the causes of elevated QTc and to rule out congenital causes of long QTc if persistent QTc prolongation was found. She was advised to avoid excessive caffeine and any potential drugs that can prolong QTc in the future, including decongestants. A four-week home telemetry was set up on discharge with the plan to place an implantable cardiac defibrillator if she developed further ventricular arrhythmias.

**Figure 3:**
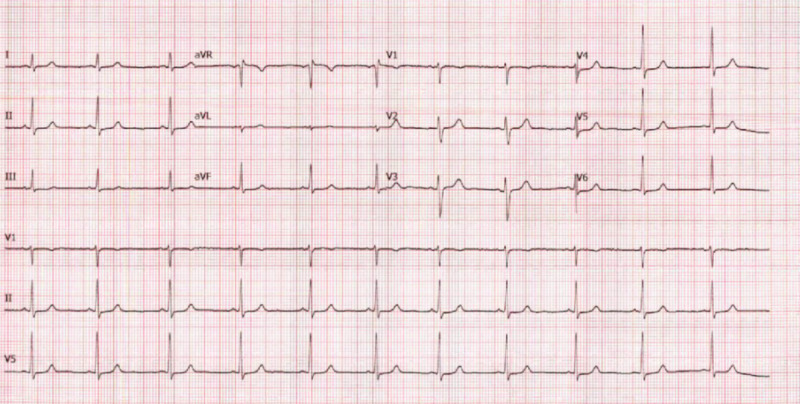
Outpatient EKG: sinus rhythm with no QT or ST abnormalities

On follow-up as an outpatient, the patient did not have any further arrhythmias. A 12-lead ECG on follow-up showed normal rhythm and QTc intervals, with normalization of previously nonspecific ST waves (Figure 3).

## Discussion

The maximum FDA-approved daily dose of loperamide is 8 mg/day for over-the-counter use and 16 mg/day for prescription use. At therapeutic doses, the systemic absorption of loperamide is minimal as it undergoes extensive first-pass metabolism via cytochrome P450 isoenzymes (CYP3A4 and CYP2C8), and due to the presence of the P-glycoprotein efflux system in the intestine tract and the blood-brain barrier, which is responsible for actively pumping the drug out of the central nervous system, penetration of the blood-brain barrier is limited. However, at supratherapeutic doses, loperamide can circumvent the p-glycoprotein efflux system, cross the blood-brain barrier, and exert effects similar to those of other opioids [1,7].

Documented cases of torsades de pointes are relatively rare, even for drugs that prolong the QT/QTc interval, and are often not reported until a large population of patients has received the drug in a post-marketing setting [7]. The mechanisms underlying the proarrhythmic effects of loperamide have been linked to the blockade of the human ether-a-go-go-related gene (hERG) voltage-gated potassium channel (also known as Kv11.1) and NaV1.5 channel. The hERG channel forms a major portion of the ion channel proteins that conduct potassium ions (K+) out of the myocardial cells and is crucial in appropriately repolarizing the cell membrane during the cardiac action potential. Inhibition of the hERG voltage-gated potassium channel results in a prolongation of repolarization that produces a widening of the QT interval on the EKG and increases the risk for torsades de pointes. Additionally, loperamide has also been shown to inhibit the NaV1.5 channel, responsible for the fast depolarization of phase 0 of the action potential, explaining the resultant wide QRS complex [6,8,9]. The reported half-life of loperamide is approximately nine to 13 hours, although longer half-lives up to about 40.9 hours have been reported with doses of 16 mg [10]. In a study reported by Swank et al. [7], some patients were noted to use concomitant drugs (CYP3A4, CYP2CB, and P-glycoprotein inhibitors) to increase gastrointestinal absorption of loperamide, decrease loperamide metabolism, or increase blood-brain barrier penetration of loperamide.

The management of loperamide-induced polymorphic ventricular tachycardia remains largely supportive and requires advanced cardiac life support. Loperamide and interacting medications should be immediately discontinued when toxicity is suspected. Naloxone has been deemed ineffective at reversing loperamide-induced arrhythmias [5]. Due to its high protein-binding characteristics, hemodialysis is less likely to remove loperamide from circulation [9]. Although the use of various agents like intravenous magnesium, metoprolol, sodium bicarbonate, lidocaine, and isoproterenol has been reported in the literature, the FDA’s review of cases found that torsades de pointes resulting from loperamide overdose did not respond well to traditional pharmacological therapy, likely due to the unique mechanisms of loperamide-induced cardiac toxicity, so cardiac pacing or electrical cardioversion may be required [5]. Intravenous lipid emulsion has been suggested as adjunctive therapy if traditional modalities fail [11]. Mechanical circulatory support may be considered for refractory cardiogenic shock, hemodynamic compromise, or recurrent cardiac arrest [9].

In June 2016, the FDA issued a warning to healthcare professionals and patients regarding the serious cardiovascular side effects associated with more than normal doses of loperamide, including prolonged QTc interval, torsades de pointes, and cardiac arrest [12]. In September 2019, the FDA limited each carton to 48 mg of loperamide and required capsules and tablets to be packaged in individual doses [13]. It is worth noting that loperamide, along with common boosters like cimetidine and ranitidine, remains available over the counter [9]. This case report adds to the cases of serious cardiotoxicity caused by misuse of loperamide despite the aforementioned measures and underscores the need for more regulatory actions for the drug. Also, loperamide is not detected on routine urine drug screening for opioids, as in our patient, who had a negative urine drug screen for opioid use. Her urine drug screen was positive for benzodiazepines, which was from receiving intravenous midazolam in the ED. Our patient also received azithromycin, a QTc prolonging agent, which, in combination with loperamide, further aggravated QTc prolongation and placed the patient at risk of torsades de pointes. The availability of loperamide over the counter and its inability to be detected on a urine drug screen enhance the importance of thorough history-taking in patients who are being administered other QTc-prolonging agents. In the current era of the ongoing opioid crisis, healthcare professionals need to be vigilant regarding the misuse of loperamide. Patients noted to have prolonged QTc intervals or torsades de pointes should be asked about the use of loperamide, and screening with interval EKGs may be considered in patients on chronic loperamide.

## Conclusions

During recent years, loperamide has been noted to be misused at higher than recommended doses to attenuate opioid withdrawal symptoms and achieve opioid euphoric effects. With the use of higher than recommended doses of loperamide, life-threatening cardiovascular adverse effects have been unmasked, including prolonged QTc, polymorphic tachycardias, torsades de pointes, and sudden cardiac death. Despite regulatory measures taken by the FDA, loperamide remains available over the counter, along with booster drugs that tend to increase the bioavailability of loperamide. This case highlights a lethal cardiac side effect of loperamide overdose: torsades de pointes leading to cardiac arrest. The case underlines the significance of the need for more stringent regulatory measures to dispense loperamide in the current opioid crisis. In patients noted to have prolonged QTc or wide complex tachycardias, a history of loperamide use should be considered.
